# Whole-Genome Sequence of *Borrelia garinii* Strain 935T Isolated from *Ixodes persulcatus* in South Korea

**DOI:** 10.1128/genomeA.01298-14

**Published:** 2014-12-24

**Authors:** Yoontae Noh, Su Yeon Kim, Yeong Seon Lee, Dae-Won Kim, Taesoo Kwon, Kyu-Jam Hwang

**Affiliations:** a Division of Zoonoses, Center for Immunology & Pathology, National Institute of Health, Korea Centers for Disease Control & Prevention, Osong Health Technology Administration Complex, Chungcheongbuk-do, Republic of Korea; b Division of Biosafety Evaluation and Control, National Institute of Health, Korea Centers for Disease Control & Prevention, Osong Health Technology Administration Complex, Chungcheongbuk-do, Republic of Korea

## Abstract

We report here the genome sequence of *Borrelia garinii* strain 935T isolated from *Ixodes persulcatus* in South Korea. The 1,176,739 bp (G+C content, 27.73%) genome consists of 1,194 coding regions, 4 rRNA genes, and 33 aminoacyl-tRNA synthetase genes. This is the first whole-genome report of a Korean *Borrelia* species isolate.

## GENOME ANNOUNCEMENT

Lyme disease is a tick-borne zoonotic disease caused by members of the *Borrelia burgdorferi sensu lato* complex. The group is composed of approximately 20 species, including *B. burgdorferi sensu stricto, Borrelia afzelii, Borrelia garinii, Borrelia valaisiana*, and *Borrelia lusitaniae* (1–4). The *Borrelia* species are mainly transmitted by *Ixodes* species, including *I. ricinus* and *I. persulcatus* in Europe, *I. persulcatus* and *I. nipponensis* in Asia, and *I. scapularis* and *I. pacificus* in North America (5–7). In South Korea, the *Borrelia* species were isolated in 1993 from *I. persulcatus* and *Apodemus agrarius* (8, 9).

The Korean *B. garinii* 935T strain was isolated from *I. persulcatus* in the northern part of South Korea (9). The strain was analyzed by Western blotting using monoclonal antibodies directed to various outer surface protein A (OspA) of other strains and the OspA of the Korean *B. garinii* 935T strain. Compared to the OspA of other isolates, that of the Korean *B. garinii* 935T strain was smaller and reacted with a unique antibody. These results indicate that the Korean *B. garinii* 935T strain is distinct in antigenicity from other *Borrelia* species strains (9, 10). In this study, the whole-genome sequencing of the Korean *B. garinii* 935T strain was performed for genetic characterization.

Total genomic DNA was purified using the G-spin total DNA extraction kit (iNtRON, South Korea) and sequenced using PacBio CLR sequencing technology on the PacBio RSII machine. The sequencing read quality was 0.8. The *de novo* assembly results for preassembly, scaffolding, and consensus polishing were analyzed by SMRT 2.1. Gene prediction was performed using Glimmer 3.02. During preassembly, 565.5 Mbp were filtered from a total of 772.6 Mbp of PacBio CLR reads. The mean read length for the genome was 10,349 bp. The genome was sequenced to 330× genome coverage.

The draft genome, comprising 1,176,739 bp with 27.73% G+C content, was annotated by the Rapid Annotations using Subsystems Technology (RAST) system server (11). A total of 1,194 coding regions were found in the genome, of which 830 (70%) were functionally annotated. The genome contains 4 rRNA genes and 33 aminoacyl-tRNA synthetase genes. The genome coding density is 86%, with an average gene length of 851 bp; 589 genes are transcribed from the positive strand and 605 from the negative strand. The annotated genome has 22 genes involved in virulence, disease, and defense, including eight genes for resistance to antibiotics and toxic compounds. Twenty genes are involved in the bacterial stress response, including five genes for oxidative stress and four genes that function to protect against osmotic stress.

RAST annotation indicated that strains *B. garinii* PBi (score, 520), *B. afzelii* PKo (score, 451), and *B. burgdorferi* B31 (score, 412) are the closest neighbors of the Korean *B. garinii* 935T strain.

This genome sequence will be a useful data set for understanding *B. burgdorferi* diversity and provide a functional platform for investigating the pathogenesis of Lyme disease.

### Nucleotide sequence accession numbers.

The whole-genome sequence of the *B. garinii* 935T strain has been deposited in GenBank under the accession number JJNU00000000 (scaffold4/0, JJNU01000001; scaffold4/1, JJNU01000002; scaffold4/2, JJNU01000003; scaffold4/3, JJNU01000004; scaffold4/4, JJNU01000005; scaffold4/5, JJNU01000006; scaffold4/6, JJNU01000007).

## References

[1] Stanek G Reiter M 2011 The expanding Lyme *Borrelia* complex—clinical significance of genomic species? Clin. Microbiol. Infect. 17:487–493. 10.1111/j.1469-0691.2011.03492.x. 21414082

[2] Wu Q Liu Z Li Y Guan G Niu Q Chen Z Luo J Yin H 2014 Genome sequence of *Borrelia garinii* strain SZ, isolated in China. Genome Announc. 2(4):e00010-14. 10.1128/genomeA.00010-14. 25035317 PMC4102854

[3] Casjens SR Mongodin EF Qiu WG Dunn JJ Luft BJ Fraser-Liggett CM Schutzer SE 2011 Whole-genome sequences of two *Borrelia afzelii* and two *Borrelia garinii* Lyme disease agent isolates. J. Bacteriol. 193:6995–6996. 10.1128/JB.05951-11. 22123755 PMC3232841

[4] Jiang B Yao H Tong Y Yang X Huang Y Jiang J Cao W 2012 Genome sequence of *Borrelia garinii* strain NMJW1, isolated from China. J. Bacteriol. 194:6660–6661. 10.1128/JB.01844-12. 23144406 PMC3497544

[5] Singh SK Girschick HJ 2004 Molecular survival strategies of the Lyme disease spirochete *Borrelia burgdorferi*. Lancet Infect. Dis. 4:575–583. 10.1016/S1473-3099(04)01132-6. 15336225

[6] Margos G Vollmer SA Ogden NH Fish D 2011 Population genetics, taxonomy, phylogeny and evolution of *Borrelia burgdorferi sensu lato*. Infect. Genet. Evol. 11:1545–1563. 10.1016/j.meegid.2011.07.022. 21843658 PMC3214628

[7] Casjens SR Fraser-Liggett CM Mongodin EF Qiu WG Dunn JJ Luft BJ Schutzer SE 2011 Whole genome sequence of an unusual *Borrelia burgdorferi sensu lato* isolate. J. Bacteriol. 193:1489–1490. 10.1128/JB.01521-10. 21217002 PMC3067611

[8] Park KH Chang WH Schwan TG 1993 Identification and characterization of Lyme disease spirochetes, *Borrelia burgdorferi sensu lato*, isolated in Korea. J. Clin. Microbiol. 31:1831–1837. 8349761 10.1128/jcm.31.7.1831-1837.1993 PMC265641

[9] Kee S Hwang KJ Oh HB Kim MB Shim JC Ree HI Park KS 1994 Isolation and identification of *Borrelia burgdorferi* in Korea. J. Korean Soc. Microbiol. 29:301–310.

[10] Kee S Hwang KJ Oh HB Park KS 1994 Identification of *Borrelia burgdorferi* isolated in Korea using outer surface protein A (OspA) serotyping system. Microbiol. Immunol. 38:989–993. 7723693 10.1111/j.1348-0421.1994.tb02157.x

[11] Aziz RK Bartels D Best AA DeJongh M Disz T Edwards RA Formsma K Gerdes S Glass EM Kubal M Meyer F Olsen GJ Olson R Osterman AL Overbeek RA McNeil LK Paarmann D Paczian T Parrello B Pusch GD Reich C Stevens R Vassieva O Vonstein V Wilke A Zagnitko O 2008 The RAST server: Rapid Annotations using Subsystems Technology. BMC Genomics 9:75. 10.1186/1471-2164-9-75. 18261238 PMC2265698

